# Early Recognition of Foreign Body Aspiration as the Cause of Cardiac Arrest

**DOI:** 10.1155/2016/1329234

**Published:** 2016-02-23

**Authors:** Muhammad Kashif, Hafiz Rizwan Talib Hashmi, Misbahuddin Khaja

**Affiliations:** Division of Pulmonary and Critical Care Medicine, Department of Medicine, Bronx Lebanon Hospital Center, Bronx, NY 10457, USA

## Abstract

Foreign body aspiration (FBA) is uncommon in the adult population but can be a life-threatening condition. Clinical manifestations vary according to the degree of airway obstruction, and, in some cases, making the correct diagnosis requires a high level of clinical suspicion combined with a detailed history and exam. Sudden cardiac arrest after FBA may occur secondary to asphyxiation. We present a 48-year-old male with no history of cardiac disease brought to the emergency department after an out-of-hospital cardiac arrest (OHCA). The patient was resuscitated after 15 minutes of cardiac arrest. He was initially managed with therapeutic hypothermia (TH). Subsequent history suggested FBA as a possible etiology of the cardiac arrest, and fiberoptic bronchoscopy demonstrated a piece of meat and bone lodged in the left main stem bronchus. The foreign body was removed with the bronchoscope and the patient clinically improved with full neurological recovery. Therapeutic hypothermia following cardiac arrest due to asphyxia has been reported to have high mortality and poor neurological outcomes. This case highlights the importance of early identification of FBA causing cardiac arrest, and we report a positive neurological outcome for postresuscitation therapeutic hypothermia following cardiac arrest due to asphyxia.

## 1. Introduction

Foreign body aspiration is uncommon in the adult population but can be associated with life-threatening airway obstruction. Symptoms can range from cough, dyspnea, choking, and acute asphyxiation leading to cardiorespiratory arrest [1]. Cardiac arrest due to foreign body aspiration is relatively less common in adults likely due to larger airway diameters [2]. Poor neurologic status, irrespective of the primary cardiac arrest arrhythmia, is the predominant cause of death among survivors of OHCA [3]. TH is indicated in survivors of OHCA from several causes with favorable outcome. However, studies specifically focusing on the outcome of TH after asphyxial cardiac arrest showed good neurologic outcomes in a very small number of patients [4, 5]. The TH maintenance times were not uniform in some of those patients. Here, we present an unusual case of asphyxial cardiac arrest secondary to foreign body aspiration with complete neurological recovery after early bronchoscopy removal and TH.

## 2. Case Presentation

A 48-year-old male was brought to the emergency department in cardiac arrest after collapsing at a grocery store. Bystanders called emergency medical services (EMS). The patient was found to have pulseless electrical activity (PEA), and EMS initiated cardiopulmonary resuscitation. During the resuscitation, the patient was bag-ventilated and intubated. Following administration of three doses of intravenous epinephrine (1 mg in 10 mL), the cardiac rhythm converted to ventricular tachycardia, and, after two shocks of 360 J, the patient reverted to sinus rhythm with good cardiac output. The total duration of cardiopulmonary resuscitation was 15 minutes.

The patient's past medical history was significant only for controlled hypertension. There was no history of smoking, illegal drug use, or other behaviors that might increase his risk of cardiac disorders. He had no family history of sudden cardiac arrest and no reported allergies. His medications included daily losartan (100 mg) and hydrochlorothiazide (25 mg).

**Figure 1:**
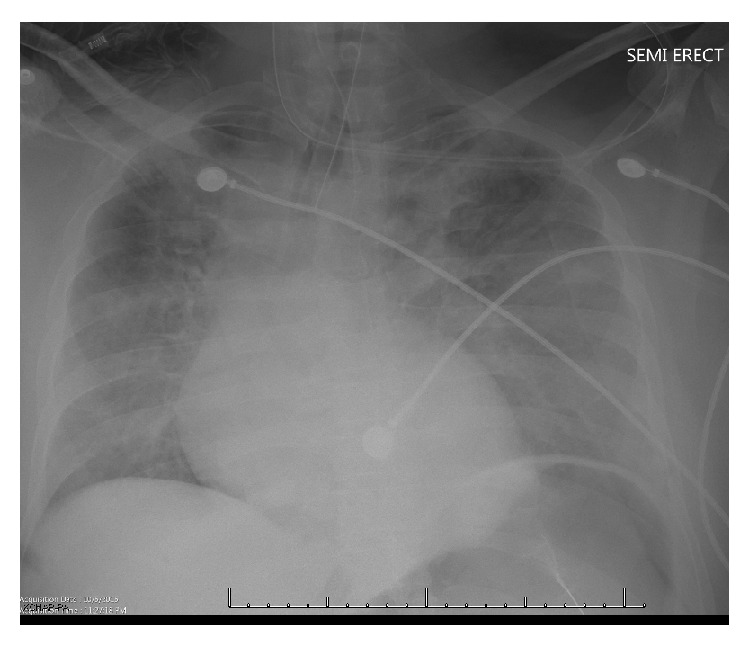
Chest radiograph shows endotracheal tube in position.

Physical exam on arrival to the emergency department revealed a middle-aged, intubated patient with a temperature of 98.6 F, pulse rate of 98 beats per minute, respiratory rate of 18 breaths per minute, and blood pressure of 98/65 mm Hg. His oxygen saturation was 100% while on a ventilator receiving 40% FiO_2_. He had no conjunctival pallor. Auscultation of the lungs demonstrated bilateral air entry with no adventitious sounds. Precordial exam revealed normal heart sounds with no murmur, rub, or gallop. Abdominal exam revealed no hepatosplenomegaly. Glasgow Coma Scale (GCS) score was three on initial assessment. Electrocardiogram showed normal sinus rhythm with T wave inversions in I, AVL, and V3–V6. Portable chest radiograph revealed an appropriately placed endotracheal tube (ETT) (Figure 1). Computed Tomography (CT) of the brain did not reveal any significant pathology. No arrhythmias were identified during cardiac monitoring. Cardiac enzymes and an initial echocardiogram did not show any ischemia.

**Figure 2:**
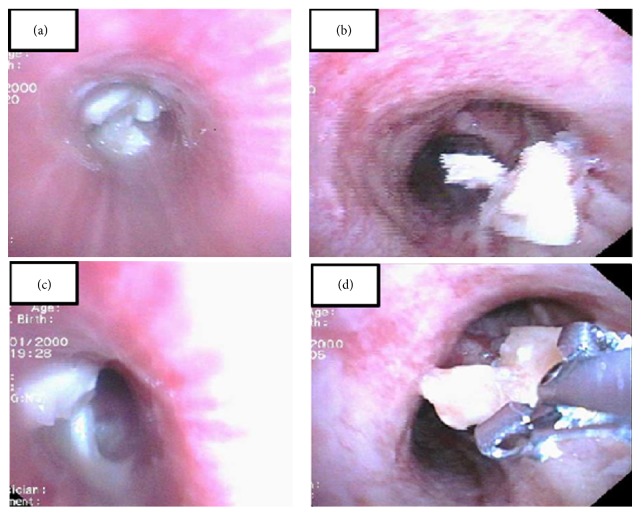
(a, c) Foreign body in the left main stem bronchus; (b) foreign body in the right main stem; (d) forceps retrieving the foreign body.

The patient was deemed a candidate for postresuscitation hypothermia and was managed according to the current hypothermia guidelines. Cardiac catheterization was performed and demonstrated patent coronary vessels. Further history was then provided by family members, who reported that the patient was choking on food prior to his collapse. In the absence of another clear etiology for cardiac arrest and with no significant arrhythmias or ischemia identified, foreign body aspiration was considered as the most plausible explanation for the patient's arrest. The patient underwent bronchoscopy, revealing a piece of meat and bone lodged in the left main stem bronchus (Figures 2(a) and 2(c)). During the retrieval process, pieces of foreign body were dislodged in the right mainstem (Figure 2(b)). Biopsy forceps were used to remove the foreign body (Figure 2(d)). Follow-up flexible bronchoscopy performed 48 hours later revealed complete removal of the foreign body and diffuse tracheobronchial mucosal edema. The patient went on to have an excellent clinical recovery and was discharged after 15 days in the hospital, with no cardiac, respiratory, or neurological sequelae.

## 3. Discussion

Foreign body aspiration (FBA) is a dangerous and potentially life-threatening event. In the United States, death from FBA is the fourth leading cause of accidental home and community deaths, but the occurrence of FBA is less common in the adult population compared to children and the elderly [6].

Vegetable matter and inorganic objects such as toy parts are also commonly aspirated objects in children, whereas adults aspirate food particles, medication tablets, dental pieces, and a variety of other inorganic substances. Choking while eating, leading to severe dyspnea followed by respiratory and cardiac arrest, may be initially misdiagnosed as myocardial ischemia. Tracheobronchial foreign body aspiration has a range of possible outcomes, including immediate resolution, recurrent pulmonary disease, recurrent pneumonia and a right lower lobe lung abscess, asphyxia, and death [7].

Aspiration of dietary or nondietary foreign bodies may occur as a result of choking while eating, in patients under anesthesia, during seizures, while a person is intoxicated, or as an accidental event while an object is held in the mouth. The risk of FBA is increased in the presence of oropharyngeal dysfunction, which may occur due to a neurologic disorder or as the result of certain sedating medications.

Aspiration events can vary widely in presentation due to factors such as patient age, degree of airway obstruction, and baseline neurologic status. These events can range from unobserved, asymptomatic episodes to complete airway obstructions with acute asphyxia and cardiorespiratory collapse. In the case of an acute airway obstruction, a conscious patient may have symptoms of aphonia, dysphagia, odynophagia, the hand-to-the-throat choking sign, stridor, facial swelling, and/or prominence of the neck veins. Clinical exam in these patients may identify absent air entry on chest auscultation, marked respiratory distress, and tachycardia [8]. In complete airway obstruction, however, asphyxiation quickly leads to hypoxia and collapse. In unconscious or sedated patients, the presentation of FBA is often less apparent, and the first sign of airway obstruction may be the inability to ventilate with a bag-valve mask. Untreated, complete airway obstruction quickly leads to cyanosis, respiratory distress, and death [9].

Making the correct diagnosis requires an awareness of the circumstances in which the aspiration event occurred, as well as consideration of subtle clues in the clinical presentation. Imaging modalities may be useful in certain cases. In addition to asphyxia and respiratory failure, other potential causes of cardiac arrest include cardiac disorders, drowning, drug use, exsanguination, and chest trauma. Lethal FBA can be avoided by immediate removal of the obstructing piece of foreign material [10], but prompt, life-saving treatment requires the diagnosis of FBA as the cause of asphyxia.

During resuscitation following an acute event, establishing a secure and patent airway is the most important goal. Available medical and surgical approaches include oropharyngeal airways, endotracheal intubation (transnasal or oral), tracheotomy, cricothyroidotomy, fiberoptic intubation, and the administration of racemic epinephrine, corticosteroids, and helium-oxygen mixtures. The addition of laser therapy, bronchoscopic dilation, and airway stenting may also be helpful [11]. Fiberoptic bronchoscopy is the diagnostic test of choice for the initial diagnosis of FBA in adults [12, 13].

Reports from the Mayo Clinic have demonstrated that the flexible bronchoscope was successful in the extraction of 100% of airway FBs in 26 children and 89% of FBs in 61 adults [14].

Rigid bronchoscopy is recommended if fiberoptic bronchoscopy fails; for foreign bodies that are centrally located or embedded in scar tissue; and for the removal of sharp objects that might cause mucosal trauma during manipulation [15].

In this case, the patient was found on bronchoscopy to have a foreign body located in the left main stem bronchus. This location contrasts with the finding in children that aspirated foreign bodies are more often lodged in the right main stem bronchus, likely due to the anatomy of the right main stem bronchus, which is shorter, larger in diameter, and at a less acute angle to the trachea than the left. The patient appeared to have initially had a complete airway obstruction, resulting in asphyxia and cardiac arrest. It is plausible that the foreign body obstructing the airway was pushed by the endotracheal tube into the left main stem bronchus, allowing for ventilation and resuscitation from the arrest.

For patients who experience out-of-hospital cardiac arrest, neurologic injury is the most common cause of death. In addition, for patients who regain spontaneous circulation, neurologic injury contributes to high inpatient mortality and morbidity rates [3]. Therapeutic hypothermia is indicated after out-of-hospital cardiac arrest for comatose, hemodynamically stable patients who experienced nonperfusing ventricular tachycardia, and ventricular fibrillation prior to resuscitation [5]. A study of therapeutic hypothermia following resuscitation from asphyxial cardiac arrest reported a 46.8% survival rate, but, in this cohort, good neurologic outcomes were reported in only a small percentage of patients (5.4%) [16].

For survivors of cardiac arrest, neuroprotection from therapeutic hypothermia has been attributed to a reduction in cerebral metabolism at reduced temperatures, estimated as a 6–10% reduction in cerebral metabolism for each reduction of one degree Celsius. Reduced brain metabolic activity prevents the progression of a cytotoxic cascade caused by free oxygen radicals, resulting in decreased cellular apoptosis between 48 and 72 hours after the arrest; decreased cerebral inflammatory responses; and improved blood-brain barrier integrity [17]. Some level 4 evidence suggests that survivors of out-of-hospital cardiac arrests from several causes may benefit from therapeutic hypothermia [18]. The target temperature for TH varies widely due to the variability in the updated recommended goal temperatures (ranging from 32°C to 36°C) in the 2015 AHA Guidelines. It is reasonable that the target temperature be maintained for at least 24 hours after achieving target temperature [19]. In this case, the patient went on to an excellent clinical outcome after receiving therapeutic hypothermia following out-of-hospital cardiac arrest with an initially nonshockable rhythm (PEA) and 15 minutes of resuscitation.

## 4. Conclusion

Tracheobronchial foreign body aspiration is a serious medical problem with clinical manifestations ranging from acute asphyxiation to insidious lung damage. This case highlights the importance of early identification and management of acute airway obstruction caused by foreign body aspiration, as well as the value of postresuscitation hypothermia for improving postcardiac arrest neurological outcomes.

## Abbreviations

FBA: Foreign body aspiration
PEA: Pulseless electrical activity
ETT: Endotracheal tube
GCS: Glasgow Coma Scale
OHCA: Out-of-hospital cardiac arrest.
